# Public Attitudes towards Cow Welfare and Cow Shelters (Gaushalas) in India

**DOI:** 10.3390/ani9110972

**Published:** 2019-11-14

**Authors:** Arvind Sharma, Catherine Schuetze, Clive J. C. Phillips

**Affiliations:** 1 Centre for Animal Welfare and Ethics, School of Veterinary Science, The University of Queensland, Gatton Campus 4343, Australia; c.phillips@uq.edu.au; 2 Faculty of Arts and Social Sciences, The University of Sydney, New South Wales 2006, Australia; vajracat@gmail.com

**Keywords:** cattle, cow shelters, gaushalas, public attitudes, welfare, India

## Abstract

**Simple Summary:**

Public attitudes towards cow welfare and cow shelters (locally known as gaushalas) in India have been little understood in the contemporary context; however, there is a plethora of historical accounts about the reverence of cows and existence of cow shelters in the Indian society. India faces an overpopulation of street cows, and the importance of the cow shelters to house these old, infertile and abandoned cows is of great interest. We conducted a survey of the attitudes of the Indian public towards cows and cow shelters in six states of India, where we found significant demographic differences in terms of age, gender, marital status, income levels, education levels, religion, level of religiosity, and place of residence. Key differences in the attitudes of the public towards cows and cow shelters across the demographic profiles delineated in this study were elucidated, which can be incorporated into initiatives to improve cow welfare in shelters. This will strengthen public engagement to successfully manage these cow shelters using modern scientific concepts of animal welfare-based management in order to perpetuate these unique institutions in a sustainable way.

**Abstract:**

Public attitudes towards cows and cow shelters in India need to be assessed in the contemporary context, as India is facing an overpopulation of street cows, leading to traffic hazards, public health issues, and pollution. We investigated the attitudes of the general public in India towards cow welfare in general and cow shelters (gaushalas) in particular. Eight hundred and twenty-five members of the public, residing in the vicinity of 54 cow shelters, were interviewed for this purpose. Their perception of animal welfare centred on animal care, cows as goddesses and mothers, and doing things properly. More than half visited a shelter daily for religious reasons. Most believed that cow shelters were the best way to manage the stray cow population and felt a community responsibility towards all breeds of cows for animal welfare reasons. Space availability for the cows was the key welfare issue voiced. Older people were more likely to identify animal welfare and culture as the main reason for sheltering cows. Better educated, wealthier, and more religious people visited the shelters most, rating religion and breeding higher as the shelter’s main purpose. Males favoured indigenous cow breeds more than females. Village respondents were more likely to consider the facilities adequate compared with country town and urban respondents. In contrast to married respondents, single people were more likely to say that they visited for leisure rather than for religious purposes. The survey indicated that the Indian community was generally supportive of cow sheltering and that visits to the shelters helped them to know that unwanted cattle were being well cared for.

## 1. Introduction

Religiously-inspired attitudes towards animals are found worldwide; however, the Indic traditions of Hinduism, Buddhism, and Jainism are particularly unique in their promotion of Ahimsa (non-harm to all living beings including animals) [1]. Religious beliefs in many parts of India have exerted a special influence on the human-animal bond, and hence the welfare of animals. The cow has an important role in the culture and religion in contemporary Hinduism in India. It represents abundance and fertility, embodying the concept of motherhood, and is the abode of 330 million gods [2,3]. Cows are also symbols of non-violence and generosity in Hindu culture; they are central to debates on vegetarianism and are associated with many Hindu gods [4]. The concept of bovine sanctity developed within the Aryan culture during the end of the Vedic period (4th century B.C.), with the first reference in the text *Chandogya Upanishad* [5].

A complicated nexus of social, religious, historical, and political factors have contributed to the widespread acceptance of this belief in the Hindu public [5]. Protests against cow killing became politicized during the Muslim invasions of the 11th and 12th centuries A.D., during India’s struggle for freedom from the British rule in the mid-20th century, and more recently with the rise of the Hindu right nationalist movement [4]. These events peaked again during the last decade, especially during the rule of the present political dispensation, linking concepts of a nationalistic identity, spiritual/caste purity and pollution, and anti-Muslim sentiment. This has resulted in vigilante cow protection groups attacking people suspected of harming cows [6]. However, not all Hindus are vegetarians or avoid beef [7], India is the second largest exporter of beef, and has one of the largest live export businesses in the world [8]. Therefore, as Staples [7] aptly writes, the picture that emerges is not straightforward, and “the (re is a) stereotypical image of India as a nation squeamish about cattle slaughter”. Certainly, in this cow contentious and highly politicized environment, the sheltering of cows in gaushalas has gained prominence once again. Due to this reverence, cow slaughter is banned in most Indian states, and the overpopulation of abandoned cows in the streets is a public health risk, traffic hazard, and an animal welfare concern [9,10].

The establishment and consolidation of the institution of ‘gaushalas’ began in the third to fourth century B.C [5,11] and persists today. A Gaushala houses cows affected by recurrent droughts and famines, as well as old, infirm, infertile, and abandoned cows. Despite economic growth in the secondary and tertiary industry sectors, agriculture is still the mainstay of the Indian economy. There are more than 5000 gaushalas and nearly 5.3 million street cows in India, according to a recent livestock census report [12]. Rapid urbanization, mechanization of farming operations, fragmentation of pastures and grazing lands, and bans on cow slaughter and euthanasia are the main factors leading to the overpopulation of the street cows in India [13,14]. The abandoned street cows cause public health risks, traffic hazards, and are a serious animal welfare concern [9,10].

The growing overpopulation challenges the capacity of gaushalas to shelter street cows and, ultimately, the welfare of cows housed in them. The majority of these shelters are located in the northern and western parts of the country, where an Aryan culture predominates, with very few in the southern states, probably due to the older Dravidian culture there [10,11]. The shelters are supported by philanthropists, temple trusts, the government, and donations from the business community and the general public. There is no uniform pattern of funding patterns for the cow shelters, and many of them suffer from limited financial support. Those located near Hindu temples and pilgrimage sites are usually well funded by devotees’ donations. Others have serious limitations, with little government support, inadequate feed for the cows, and poor infrastructure. Despite these problems, the cow shelters manage to sustain themselves, but it is not clear to what extent they garner popular public support, nor what the Indian public attitudes towards gaushalas are.

Public attitudes are the drivers of change and can be determined by social science research revealing societal issues and concerns. Beliefs guide public attitudes, and attitudes determine public behaviour as citizens [15]; understanding both attitudes and beliefs are therefore of prime importance for coordinating and guiding improvements in the welfare of animals [16]. Beliefs and understanding of animals by any society is species specific, especially the extent to which it is given priority and resources [17]. There has been significant research conducted on the public attitude towards farm animals, and specifically cows, in Europe, North America, and Australia [18,19,20,21,22,23]. However, no study has exclusively focused on public attitudes towards cattle welfare in India, a country that has the world’s largest cattle population [24] and some apparently quite unique perspectives on managing unwanted cattle.

Three types of motivations have been proposed for the response of the public toward animals: self-interest, empathy, and values about the status and nature of the animals [25]. While religion, culture, and socio-economics moderate public attitudes towards animals [26], an animal’s nature and its characteristics also influence public attitudes [27].

Attitudes affect the way animals are treated and, according to the Theory of Planned Behaviour, the intent of an individual to behave in a certain manner is a prerequisite for the implementation of a particular behaviour [28,29]. Self-evaluation of the behaviour (attitude), a belief that the behaviour can be realized (perceived behaviour control), and the opinions of individuals whom the person considers important (subjective norm) determines the intent of performing a behaviour [28,30]. A study in the USA found that love for animals, as well as economic and practical considerations, was the primary motivational factor in the attitude of the American public towards animals [31].

Understanding the attitude of the public towards animal welfare is important both at an individual level as well as at a societal level. Policy formulation and legislation to improve animal behaviour are influenced by public attitudes and how they are changing [17]. Scientific studies providing evidence to improve welfare will be inconsequential in bringing about changes unless they are supported by positive public attitudes and cultural values [16]. Attitudes towards animals develop early in life but are also transformed during adulthood, which justifies widespread public education [15,32]. Cultural practices and attitudes towards animals can change over time, but they may also persist, reflecting historical traditions [16]. Although there have been studies on the attitudes and knowledge level of Indian farmers towards animals and animal welfare [33,34,35,36], to date, no study has assessed public attitude towards cows and cow welfare in gaushalas.

Therefore, the aim of this study was to assess the public attitudes surrounding cow welfare and cow shelters in India. It was hypothesized that the attitudes of the public towards cows and cow shelters would be influenced by key demographic factors and that this would influence behaviour. It was also anticipated that due to the rapid urbanization and modernization of Indian society, the spiritual symbolism of the cow, its special status, associations with the goddess, and people’s interaction with cows in shelters might have waned or transformed.

## 2. Materials and Methods

In studying the Indian public’s perception of cow welfare and cow shelters, we considered a public perception as a social normative derived from knowledge explained and shared socially [37,38]. A quantitative questionnaire was designed that addressed (1) the public’s understanding of the cow shelters and (2) the public’s attitude towards cow shelters and cow welfare in India. Socio-demographic questions were included to further elucidate the contemporary perception and attitude of the Indian public towards cow shelters and about cow welfare. The questionnaire was designed considering the scarce literature on public knowledge and attitudes towards cows in India [11,16,39,40].

At the same time as visits to shelters were made in six states of India (Gujarat, Maharashtra, Rajasthan, Punjab, Haryana, and Himachal Pradesh) [9], a face-to-face public survey was conducted in the vicinity of each of the 54 shelters from December 2016 to July 2017. Initially, we conducted a pilot survey by randomly selecting 15 individuals near the first cow shelter visited in the state of Himachal Pradesh. Following the pilot survey, a minor adjustment was made in the language and order of questions to avoid any possible bias or leading responses. During each shelter visit, people were approached to request an interview in areas around shops, in fields, and by knocking on houses door to door, in order to obtain a broad spectrum of views from those who resided within a 1 km radius of the shelters, and who, therefore, were likely to have experience and knowledge about cow sheltering. Qualifying factors were that people should be 18 or over, that they resided within the 1 km radius of the cow shelter, and that they should not be working or have worked in the cow shelter. This generated a total of 810 responses, to which the 15 from the pilot survey were added. Each interview lasted about half an hour. The University of Queensland Institutional Human Ethics Committee granted the Human Ethics Clearance (approval number 2016001243).

### Questionnaire Design

The questionnaire focused on the public knowledge and attitudes (defined as the psychological tendency expressed after the evaluation of a particular entity with some degree of favour or disfavour [41]) towards cow shelters (termed gaushalas in India) and cow welfare. Initial questions addressed their attitude towards gaushalas, how often they visited gaushalas (once a day, once a week, once a fortnight, once a month, once in 6 months, once a year, less than once a year, or never visited); why they visited them (for religious reasons, feeding cows, educational reasons, examining welfare standards, leisure and enjoyment from seeing cows, to buy cow products, or other reasons); to rank the importance of different reasons for the establishment of gaushalas, one being most important to six being least important (for cow welfare, production and sale of milk, breeding of cows, attracting funds from rich people, religious purposes and making a profit from the sale of milk, manure, cows and calves); the best way to deal with unwanted cows (keep them in gaushalas, let them roam the streets, export them to neighboring countries, or slaughter them); whether they preferred local Indian breeds of cows over cross breeds or exotic breeds; community responsibilities to stray cows, and to what extent whether the respondents felt it important that cows should be housed in gaushalas. The questions also covered the extent of agreement on the reasons for keeping cows in gaushalas (for tradition/culture, for animal welfare, breeding, or milk production). Importance and agreement questions were rated on a five-point scale.

Further questions related to the particular gaushala located near the respondents’ residence: (a) the maximum number of cows for acceptable animal welfare (<50, 50–100, 101–150, 151–200, 250, 500, 1000, or according to space availability), (b) agreement that the gaushala gave adequate shelter, food and water, freedom to move and socialise, bedding, flooring and opportunities to lie down, veterinary care, and humane treatment for the cows; (c) whether they supported or had any issues with their local gaushala. An open-ended question was also posed to each respondent: “What do you understand by the term ‘welfare of cows’?” Finally, demographic questions were included to determine the respondents’ gender, age, religion, religiosity level, ethnicity, education level, marital status, number of children, income, place of residence, and whether they grew up with cows nearby. Answers to all these questions were self-declared except for place of residence, which was classified as urban, suburban, country town, or village by the research team and confirmed by the shelter manager.

## 3. Statistical Analysis

Data were initially collated, and controls were employed to remove data errors, using a statistical package for the analysis (Minitab^®^ version 17.1.0, Minitab Ltd., Pennsylvania State University, State College, PA, USA). A series of chi-square tests were conducted to examine the differences in response patterns for questionnaire items based on demographic variables. Independent variables were categorical and included gender, age, religion, religiosity, ethnicity, education level, marital status, number of children, income level, and place of residence. The dependent variables were either ordinal, such as frequency of visits to a gaushala, or nominal, such as their reason for visiting a gaushala, reason for and importance of establishment of the gaushalas, what was best for unwanted cows, preference for a specific cow breed, and responsibility of the community to specific breed types. Some of the ordinal dependent variables in some items in the questionnaire consisted of the level of agreement with the given items, from one (strongly disagree) to five (strongly agree). Cross tabulations between demographic variables and agreement level and opinion items were analysed by Chi-square analysis of association, ensuring that no more than 20% of the expected counts were <5, and all individual expected counts were ≥1 [42,43]. Logistic regression analyses (either binary, nominal or ordinary as appropriate to the response structure) were used to analyze the effects of demographic variables on attitude questions. Some of the data were dichotomised for analysis in cases where the distribution was highly skewed (religion—Hindus and others; ethnicity—Indo-Aryans and others; support to shelters—yes or no). Public behaviour (frequency of visiting shelters) was also analysed against public attitudes towards gaushalas and the cows using ordinal logistic regression. Logistic regression analyses were used to assess the significance of the relationships between respondent demographics (categorical independent variables) and the distribution of Likert scale responses for each attitude questions (continuous dependent variable). An iterative reweighted least squares algorithm with a logit link function was used in the model. All models achieved convergence. Referent groups were selected as those with the most responses. All probability values were considered significant at *p* < 0.05.

Thematic analysis of the open-ended question about what the respondent understood by the term ‘welfare of cows’ was conducted using NVivo Pro 12 software (NVivo qualitative data analysis software; QSR International Pty Ltd. Version 12, 2018, https://www.qsrinternational.com/nvivo/nvivo-products/nvivo-12-plus). The different responses were analysed, and the main trends extracted. A manual inspection of the source data was conducted, and the word frequency and word cloud function identified themes to the responses. Through NVivo, words were chosen for analysis based on the total number of times they appeared. However, conjunctives (such as ‘and’) and words that drew no relevance or usefulness to the theme of the study were excluded manually from the output and the analysis repeated.

## 4. Results

In the multivariable analysis of the demographic data, only the significant results are reported. However, in the descriptive analysis of the data, all the responses to the questions have been reported as numbers and percentages.

### 4.1. Respondents Demographics

Completed questionnaires were obtained from 825 respondents, with approximately equal gender representation. The response rate in this study was 80%, as on an average three out of every 15 people per shelter we approached declined to participate in the survey. The median age bracket was 36–45 years of age, slightly older than the mean Indian age (Table 1). The majority of the respondents were Hindus (96%), with very few Muslims (2%) and Sikhs (2%), both being less than the national average. Nearly all (98%) were of Indo-Aryan ethnic descent, which is higher than the national demographic. Most respondents felt they were religious, either moderately (50%) or very (47%). Just over a quarter did not attain a grade 10 educational level, 36% completed grades 10 or 12, 14% succeeded to a university graduate and 13% had no formal education. Educational levels were higher than the national average. Most respondents were married (85%), and most had two (38%) or three (21%) children. The most commonly reported (26%) annual income level was 100,001–500,000 INR (USD1461–7300). Most respondents (70%) resided in villages, and 22% in urban areas, less than nationally. Nearly all (93%) had grown up in close contact with cows during their childhood, and 99% were aware of the existence of their local gaushala.

#### Perceptions Regarding Gaushalas and Abandoned Cows

Almost one-half of the respondents reported visiting their local gaushala regularly, once a day or once a week (Table 2). The most common reason for visiting the gaushalas was religion, followed by the examination of cow welfare standards, and then feeding the cows. Almost all indicated that sheltering abandoned/unwanted cows in gaushalas was the best solution to manage unwanted street cow populations. The majority had no favourite breed of cow, but one third favoured local Indian cow breeds, and most said that the community has equal responsibility towards all cow breeds.

Nearly all participants said it was important for cows to be sheltered in gaushalas (96%), usually for animal welfare reasons, and most believed that this was culturally important. Most disagreed with using gaushalas to breed cows or for milk production purposes.

The majority of the respondents thought that the available space for cows in the gaushala was the key welfare issue; however, most agreed that their local gaushala provided adequate resources for the cows—shelter, adequate food and water, freedom of movement and opportunities for socialisation, bedding, floor space, and opportunities to lie down. Most agreed that the cows in their local gaushala were treated humanely by the workers and that there was adequate provision of veterinary care. Nearly all actively supported their local gaushala through voluntary work, donations, and moral support, and only a small minority said they had issues with their local gaushalas, which were mainly the problems of flies and mosquitoes, offensive odours, and waste management.

### 4.2. Demographic Effects

#### 4.2.1. Age

In relation to the purpose of gaushalas, the youngest age group (18–25) of respondents were more likely to rank animal welfare either very high or very low, and also rank breeding lower, compared with the older age groups (see Table 1 for the number of respondents in each category). Those in the 46–55-year-old age group were more likely to rank milk sales higher than older or younger respondents. The oldest age group were more likely to rank attracting funding higher, and the 26–35-year-old respondents were more likely to rank it lowest (Table 3). The youngest age group was more likely to rank earning a profit at a higher level than older age groups.

When asked the reason for keeping cows in gaushalas, older people (>55 years) were more likely to strongly agree that it was for animal welfare and cultural traditions than younger people (<36 years). Young people (<36) were more likely to be neutral about whether cows had adequate shelter.

#### 4.2.2. Educational Level

As education level increased, so did visit frequency, and the respondents were more likely to rate religion and breeding as the most important the purposes for establishing gaushalas and less likely to rate animal welfare and milking highly (Table 4). Similarly, they were more likely to disagree that milk sales are an important reason for keeping cows in gaushalas, and they were more likely to say that bedding and lying space, humane treatment of cows, and veterinary treatment were inadequate. As education levels increased, respondents were less likely to cite examining cow welfare as the reason to visit cow shelters.

#### 4.2.3. Gender

Men reported visiting the shelter more often, weekly, whereas women said that they only visited approximately monthly (Table 5). However, women believed the establishment of gaushalas to be slightly more important for the welfare of cows. Women ranked milk sales and breeding cows as reasons to keep cows in gaushalas higher than men. Men agreed more than women that cows in gaushalas have adequate freedom to move about and socialise with other cows. When asked to choose one reason for visiting the gaushala, males (15.7%) were more likely than females (8.5%) to say that they would visit to examine cow welfare standards, compared with visiting for religious reasons (M 62.9%, F 66.6%) (OR, 2.70, CI 1.38–5.29, *p* = 0.004). Males (40%) were more likely than females (26%) to say that their favourite type of cows were local Indian breeds.

#### 4.2.4. Income

As income level increased, the frequency of visits to the cow shelters increased. High income respondents ranked the breeding of cows higher as one of the important purposes of the gaushala (OR 1.12, 95% CI 1.04–1.21, *p* = 0.003). Middle income categories were less likely to say that they visited a gaushala to feed the cows (9.3%) than they were likely to say that it was for religious purposes (16.9%) (OR 1.15, CI 1.0–1.33, *p* = 0.05).

#### 4.2.5. Religion Effects

Hindus said that religious purposes of gaushalas were more important and making a profit was less important, compared with non-Hindus (Table 6). Hindus were also less likely to agree that milk sales were a reason for keeping cows in gaushalas and less likely to agree that shelter and bedding, flooring, and lying provisions were adequate in the gaushalas.

#### 4.2.6. Religiosity Effects

People who that said that they were very religious were more likely to visit daily and less likely to visit infrequently. They were less likely to rate profit as the most important purpose for gaushalas, and more likely to rate religious purposes as important (Table 7). They were also more likely to say that shelter, freedom to move around, bedding, flooring and lying down, humane treatment, and veterinary care were adequate. The number of respondents who visited for religious reasons increased with self-declared religiosity, and visiting for other reasons, for to feed the cows, to examine cow welfare standards, become educated, or for leisure, decreased with increasing religiosity.

#### 4.2.7. Place of Residence Effects

Urban respondents said they visited more often than village respondents (Table 8). Village respondents said that gaushalas were more important for cows than did country town respondents. Suburban, urban respondents, and to a lesser extent, village respondents, thought that animal welfare and religion were more important purposes for gaushalas, and milk sales, breeding cows, attracting funding, and earning a profit were less important, compared with country town respondents. Village respondents were more likely to consider shelter, freedom to move about and bedding, flooring, and lying down adequate compared with country town respondents, and more likely than urban respondents to consider shelter and bedding/flooring/lying down adequate. Suburban respondents were less likely than urban respondents to cite leisure as their reason for visiting compared with for religious reasons.

#### 4.2.8. Marital Status

In contrast to married respondents, single people were more likely to say that they visited gaushalas for leisure rather than for religious purposes (OR 6.47, CI 1.56–26.84, *p* = 0.01). Single people (14%) were less likely than married people (35%) or widowers (40%) to prefer Indian cattle breeds to all breeds (OR 4.07, 95% CI 1.94–8.49, *p* < 0.001). There was only one significant effect of the number of children—as it increased, the sale of milk was ranked as a more important function of the gaushalas (OR 0.84, CI 0.73–0.97, *p* = 0.02).

### 4.3. Influence of Attitudes towards Cows to Frequency of Visits to Gaushalas

**Figure 1:**
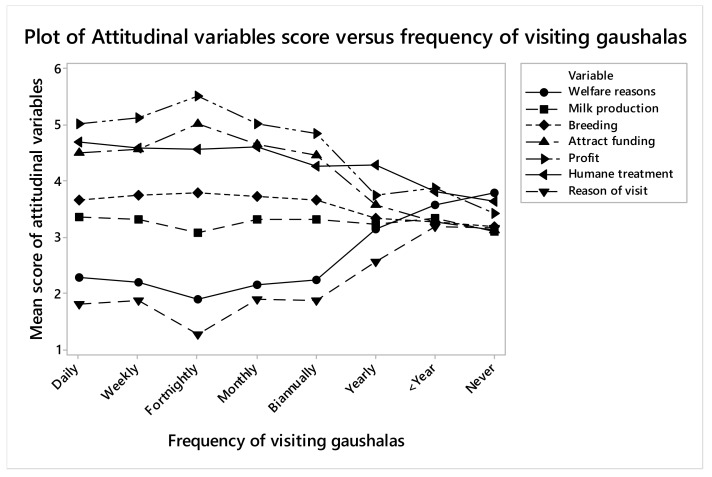
Relationship of various attitudinal variables with the frequency of visits of the public to the gaushalas.

People who frequently visited gaushalas were more likely to say that cows were humanely treated (OR 1.45, CI 1.10–1.89, *p* = 0.007) than those who rarely visited them (Figure 1). Respondents who visited daily were more likely to cite welfare as the reason for establishing gaushalas (OR 1.31, CI 1.08–1.58, *p* = 0.005) than those who visited fortnightly, but respondents who visited monthly or less frequently were again more likely to cite welfare as the reason for establishing gaushalas. Respondents who cited profit-making as the reason for establishing gaushalas were likely to visit gaushalas more frequently (OR 1.28, CI 1.05–1.57, *p* = 0.01), which could be for buying milk as most of the respondents have ranked sale of milk as the second most important reason for establishing gaushalas. Respondents who ranked religion higher as the reason of visit to gaushalas were more likely to visit them frequently than the ones who cited other reasons to visit (OR 0.90, CI 0.82–0.98, *p* = 0.01). People who rarely visited the gaushalas did not have clear reasons to visit them.

### 4.4. Qualitative Assessment

**Figure 2:**
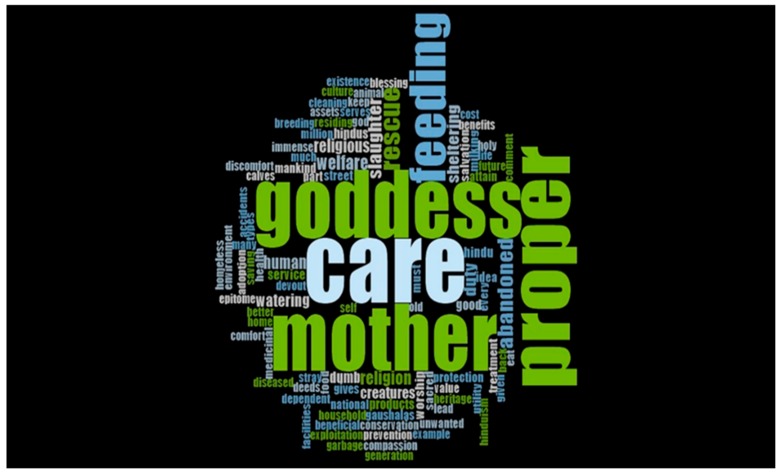
Word Cloud for the question, ’What do you understand by the term ‘welfare of cows’?’

All respondents answered the following open-ended question: What do you understand by the term ‘welfare of cows’? One-hundred-and-forty-seven word-frequencies were developed in response to the answers (Table 9). Words that were detected >10 times were as follows: care (*n* = 369), goddess (316), mother (314), proper (313), feeding (176), rescue (71), abandoned (49), slaughter (34), welfare (29), duty (27), religion (26), sheltering (26), human (23), religious (22), watering (20), creatures (15), dumb (13), Hindu (10), and worship (10). The word cloud (Figure 2) generated emphasized the almost equal and predominant importance of four related concepts: care, goddess, mother, and proper.

## 5. Discussion

This was the first study undertaken to investigate the attitudes and beliefs of the Indian public about gaushalas and about cow welfare. The aim of this study was not only to explore public beliefs about gaushalas and cow welfare but also the factors associated with these beliefs. Additionally, this study aimed to investigate preferences for the different cow breeds, the suitability of gaushalas for managing unwanted street cows, and factors associated with the preferences for the management of cows in gaushalas.

The response rate in this study was higher than other animal welfare surveys [19,45], giving confidence that it accurately depicted the attitude of the communities surrounding these gaushalas, with little non-response bias [46]. The demographic profiles of the samples in this study appear to be similar to the national profile of the population in some respects; however, the proportion of Hindus was greater, probably because gaushalas were mainly established in Hindu-centric communities. The ethnicity of most of the respondents was Indo-Aryan because the area of the study (north and north-western states of India) is predominantly composed of this ethnic group. Similarly, few of the respondents were urban as most of the gaushalas studied were located in the villages and country towns. It is possible that the response rate was higher due to the data collection method (face to face interviews), compared to using the internet or phone calls. This became relevant as the majority of the respondents had limited internet access and low literacy levels. Face to face interviews take more time, but they are better at obtaining a representative sample and can use a flexible questionnaire construction and design [47], even though web surveys do not always have low response rates [48]. The random selection of respondents from the general public who were not aware of the nature of the survey, and with the preconditions that they were not employed in the nearby cow shelter and yet living within a one km radius of the shelter, is unlikely to have created any bias in this study. These selection criteria were important for eliciting the opinions and attitudes of the public who were neutral but also lived near enough to a shelter to be aware of them and their conditions. Additionally, the recent highly politicized cow conservation movement may have contributed a positive bias on responses in the survey.

The median age group of the respondents of this study (36–45 years) is higher than the national average of 27 years. This might be due to the younger age groups being at work or college during the day. Moreover, as most of the respondents were from rural areas, younger people may have lived away from home, for work, and only occasionally return to meet elders [49]. There might be an overlap in the age groups in this context, as in India persons aged between 15–59 years are supposed to form the working age population [50]. However, 70% of our respondents were rural, and the age group of 45–65 years primarily constitute the agricultural farmers living in the rural areas working on their traditional land.

### 5.1. Perceptions about Shelters and Abandoned Cows

There were consistently positive responses to gaushalas across multiple districts in six states of India, where the majority of people report visiting regularly and contributing towards the running of the shelters. This finding suggests that the gaushalas and cows are an important part of the community in these areas and have become integrated into their social and spiritual life. While this important aspect of Hindu spiritual life has been reported in the literature [51], the extent to which gaushalas are integrated into the fabric of the community has not been explored in depth by social scientists and anthropologists and would be an important focus for future research, particularly given the recent prominence that cow protection movements have come to occupy in the current political climate in India.

Cows are venerated as goddesses by Hindus, and all religious occasions in Hindus households have worship of the cow as an important aspect of the ceremony right from the birth of a child to the death of an individual. Festivals like *Gopashtami* and *Govardhan* puja are cow centric occasions which underline the sacred cow concept in Hindu society, as people visit shelter homes and make donations for the welfare of cattle in shelters [11]. Circumambulation of the cow, similar to the one done by Hindus around their temples, is considered auspicious and equivalent to a pilgrimage to a sacred Hindu city [51].

Despite the arguments against the economic viability of the cow shelters and the cows housed in them, the Hindu society holds the welfare of cow as a duty towards the religion, which professes the concept of ‘Ahimsa’ or non-violence towards all forms of life. Though this motivation comes from religion, the sheltering of cows is an example of preventing animal wastage through active public support.

Regular visits to cow shelters for religious reasons reflect the veneration of cows in the daily life of these members of the Indian public and confirms the reverence of cows in Indian society [51]. This reverence for the cow was further confirmed by the absence of choice of any particular breed of the cow (exotic or local), and the fact that many in the community (65%) responded that they felt responsible for the cows’ welfare. A majority of the respondents favoured community responsibility for all abandoned and street cows, again reflecting the spirituality ethic embedded in Indian society towards the welfare and protection of the cows [10].

The disagreement of the public that the cow shelters were meant for breeding and milking purposes in this study confirms the ascribed Hindu values and belief system in which sheltering of the cows has a religion-based welfare motivation, though, in the post-independence era, economic returns from shelters were encouraged by the Government. [51]. Hence, the cows were utilized for milk, draft, and manure, as well as being cared for until they died of natural causes in gaushalas. This might be due to greater awareness of the public about the importance of cow shelters in the contemporary context, as limited space allowance was identified as a welfare issue in this study, suggesting that respondents believed that there should be adequate space for all cows. Most of the respondents (>82%) expressed agreement that the cows in the shelters provided a good level of welfare, similar to that described by the RSPCA’s ‘five freedoms of animal welfare’, with strong agreement that cow shelters provide adequate shelter, food and water, humane treatment, and adequate veterinary care. Additionally, active volunteering and very few issues raised by the public indicates that they were satisfied with the adequacy of cow welfare in the shelters. The responses reflect a loyalty towards their local cow shelter, supported by the fact that half of the respondents visited the shelters daily or at least weekly. However, the knowledge levels of the public about cow welfare were not assessed, which limits the validity of the conclusion that the welfare of the cows was adequate in the shelters.

### 5.2. Demographic Analysis

#### 5.2.1. Age and Number of Children

During this survey, the younger age groups spent less time per shelter visit and had less social interaction. They also ranked the welfare of cows at either end of the spectrum, either very high or very low, which may be due to a lack of interest or time spent to accurately observe welfare. They also ranked breeding lower, as traditionally cow shelters have not served this purpose. The older generation witnessed the times when breeding was one of the prime purposes of the gaushalas, and accordingly, they ranked the purpose of breeding higher. Similarly, older people tend to donate regularly to support the cow shelters, which could be the reason why they ranked attracting funding higher than younger people. The older generation listed animal welfare and cultural tradition as the reasons for keeping cows in shelters more, probably because they have witnessed the sacred cow social movements in the post-independence era when the government actively supported the opening of cow shelters [52].

The utility of cow shelters to feed the rural poor through the sale of milk could be the reason that milk sales were ranked higher as a function of cow shelters as the number of children increased in a family. The finding that respondents with children at home agree with the shelter selling milk but disagree that profit-making is an important reason to establish shelters is an interesting contradiction and invites further research. However, the sale of dairy products and dung by the cow shelters has been the traditional practice to cover the running costs [53].

#### 5.2.2. Educational Level

The frequency of cow shelters visits increased with higher educational levels, and those visits were mainly for religious reasons. In general, as educational levels increase, so do income levels [54], and disposable income allows people the freedom, mobility, and time to pursue leisure activities such as frequent gaushala visits. Moreover, education tends to make citizens more discerning and could have empowered such respondents in this study to objectively assess the availability of food, water, space and treatment for the cows, and to voice concerns over these aspects of comfort and welfare.

However, it was strange to find that as the education level increased, examining cow welfare as the reason to visit cow shelters decreased. By contrast, in Europe, religious beliefs and participation in religious practices have decreased with rising education levels and living standards in Europe in the 20th century [55,56]. A negative relationship has been observed between religion and education [57]. However, religion plays an important role in daily life in developing and emerging economies, as religious beliefs and involvement run deeper in these communities [55,58,59].

#### 5.2.3. Gender

The neutrality of female respondents about the cow’s freedom of movement and opportunity to socialise with other cows is intriguing, as most of the animal husbandry work at home in India is done by women. There is a general perception and published evidence that women have more sensitivity and empathy towards animal welfare and animal issues [35,60,61,62,63], and women are found to be more sympathetic towards animal welfare and sensitive to animal suffering [60]. However, major gender inequalities exist in India and women’s level of confidence to express their opinions about animal husbandry has a strong correlation with socio-cultural elements from their place of residence [64]. Male domination due to the patriarchal Indian society may inhibit women from expressing their opinions freely, as traditionally, men are in the position of power [65,66]. However, cross-cultural studies have suggested that in countries with a low gender-inequality index, women express their views on animal welfare more freely [63], being more supportive than males [35,60,62]. In India, the gender empowerment index value is low (0.53), with a ranking of 125th in the world [67], which suggests that women would not feel empowered to express their animal welfare concerns.

In the Indian context, males are given more authority and may enquire more into the affairs of the local cow shelter than females, who tend to be restricted to the household duties and have lesser opportunity and time to closely monitor the welfare of cows in the shelters. This could explain why men said the main reason to visit shelters was to examine cow welfare standards compared to women, who cited religion as their main reason. The cultural feminist theory suggests that women tend to make moral judgements more on the basis of relations than the general view of what is right or wrong [63], which could explain women making more critical judgements about the provisions to the cows in the shelters.

Males favoured the local Indian breeds of cows more than females. In a patriarchal Indian society, there may be discrimination against the crossbred or exotic cattle from being sheltered in cow shelters and protected by law, as they are considered inferior to the native Indian breeds [68]. Females hold a more romantic view of animals with affection and concern for them, whereas males favour the Darwinian approach, where nature is controlled and exploited [69]. The male preference for the local Indian cow breeds indicates that they are spirited nationalists, whereas women, despite being equally nationalistic, might identify a broader perspective of motherhood in cows irrespective of their breed. The patriarchal Indian society and households [66] could, therefore, be the driver of such attitudinal differences between the genders.

#### 5.2.4. Income Level

The increased visits to cow shelters with increasing income levels could be due to the availability of more time compared with those in the lower income groups. Feeding and worshipping the cow is considered to attract more wealth in Hindu mythology because the cow is also believed to be an incarnate of the Hindu goddess of wealth “Lakshmi” [5]. Similarly, the breeding of cows is also equated with growth in wealth [70], and this could be the reason why breeding was ranked higher as a function of the cow shelters by high income earners.

Those in middle income categories may have had a strong desire to uplift their economic status, and their visits to the cow shelters being for religious reasons rather than to feed cows might have been due to the Hindu belief that one can attract wealth through the worship of cows. This is a deeply ingrained in Hindu philosophy, together with the tradition of non-violence and reverence for the cow [39].

#### 5.2.5. Religion

Non-Hindus in this study lay more emphasis on the adequacy of sheltering, bedding, and flooring, indicating that they viewed the cow shelters through a prism of cow welfare and comfort rather than from a religious angle. However, they represented just 5% of the sample, which limits any conclusions. However, studies have shown that eastern religions (Hinduism, Buddhism, Confucianism) induce less religiosity than Christianity and Islam, and within India, average religiosity scores of Hindus is significantly lesser than Muslims [55].

#### 5.2.6. Religiosity

More religious people took a very optimistic view on the existence and performance of the cow shelters. They frequented the cow shelters more and attached more religious importance to the cow shelters rather than for economic reasons. Their overwhelming faith in religion and their local gaushala might be the reason they did not see, or rather ignored, the inadequacies in the welfare levels of the cows. Religiosity has been a factor influencing social behaviours and is also affected by the precise religious affiliation, some demanding more than others [71]. Since the majority of the respondents were Hindu, a religion which traditionally attaches importance to cow shelters, this was reflected in the strength of religious beliefs (religiosity) expressed by the adherents.

Self-declared, very religious respondents visited the cow shelters for religious reasons rather than for examining the welfare standards, becoming educated or for leisure. These visits to the cow shelters follow a ritualistic pattern in Hindu society that might be an individualistic passion towards religion or sometimes ordained by religious priests to bring about abundance in life, personified by the mythical ‘*Kamdhenu*’ cow, representing abundance and fertility [2]. Studies have shown high correlations between religiosity and low animal welfare concerns [19]. It could be that a deep faith in the Hindu religion and its cultural traditions might override other reasons for visiting the cow shelters. However, a limited study in the United States [72] found a curvilinear relationship between religiosity and support for killing animals, as very religious or irreligious participants supported animal killing more than moderately religious participants.

#### 5.2.7. Place of Residence Effects

There were varied and sometimes conflicting results for this category. Due to the rapid pace of urbanization and changing social, economic, and spatial demographics of modern India, extensive and recent sociology studies into these changes, which may better explain some of these findings, are few [73,74,75]. Rapidly expanding country towns in India are inhabited by low income working class or middle class citizens who cannot afford to reside in the urban areas due to financial constraints [76]. The higher literacy levels in urban and suburban areas as compared to rural areas in India [77] could be the reason for this awareness of animal welfare and their objectivity.

Suburban people were observed to subscribe to a utilitarian view about the cow shelters, as milk production, breeding of cows, attracting funding and earning profit were the ranked higher than cow welfare and religion as reasons for establishing cow shelters. During the field surveys, we observed gaushalas supplying subsidized milk to suburban people, and this may influence their views about the utility of shelters.

The higher rank of animal welfare and lower rank of profit-making and attracting funding by urban and sub urban respondents than rural ones in this study could be due to greater awareness and frequency of visits by these residents to the cow shelters. Urban dwellers also pointed out the lack of proper sheltering, bedding, and floor space. High awareness levels of the residents in the urban and country towns about cow welfare could be the reason for this perception.

Suburban residents comprise the working class, which might be religious but have less time for leisure than the affluent urban residents. This could be why suburban residents visited cow shelters more for religious reasons than for leisure.

#### 5.2.8. Marital Status Effects

Indian single people are more likely to occupy the younger age group in this study, and therefore, we would expect there to be similar correlations between unmarried and younger age effect. Interestingly, however, this was not the case. Single people, in general, have fewer obligations and more leisure time than married people, which could be why they rate leisure as the purpose to visit gaushalas.

Since the 1950s, exotic cow breeds were introduced into breeding programs across the country. The very older age groups witnessed the gradual transition of genotype from indigenous to exotic breeds and may hold a sentimentality towards the local breeds of their youth

The reason why single people visited cow shelters more for leisure as compared to married people who visit for religious reasons could be that there is more of a social obligation on the families to follow cultural/religious traditions and duties than single people. Visiting cow shelters for religious reasons could be a social and community need in close-knit Indian families [78]

Single people rated all types of cows as equal in contrast to married people and widowers who rated local Indian breeds higher. Single people in this study were mostly younger in age and seem to have a broader view of animal welfare, as evident in the earlier results of marital status effects in this study. They might be less sensitized to the sacred cow concept and view universality of compassion towards all living creatures.

### 5.3. Influence of Attitude towards Cows to Visiting Frequency to Gaushalas

The results clearly showed that more frequent visitors to shelters cited higher levels of religiosity, ranked welfare and profit-making as the reason for establishing the gaushalas, and strongly said that cows were treated humanely. Interestingly, those that visit monthly or more also cite welfare as the reason to establish shelters. Attitudes and personality explain human behaviour, [28] and, in this study, a positive correlation was found between attitudes and behaviour, like visiting shelters. The positive influence of human attitude on behaviour towards cows has been researched [79,80]. Such attitudes might indirectly affect and influence the welfare of sheltered cows.

### 5.4. Qualitative Assessment

Results of the qualitative analysis indicated that cows still hold a sacred position of the ‘Mother Goddess’ in Indian society, and this is the reason for taking care of them. The word query and count results reflect the concern for the abandonment and slaughter of cows. The care of cows through rescue from slaughter and the proper feeding for their welfare was perceived as a duty of the adherents to the religion.

## 6. Limitations of the Study

The random selection of respondents in this study significantly reduces the potential for selection bias. The selection of only those respondents who lived near the cow shelters might induce a bias, but it was intended to get information about the day to day working of the cow shelters from persons who had had the opportunity to visit them. Majority of the people living near the gaushalas were Hindus, and this skewed distribution of religion in our dataset might have induced some bias in our study.

There is a possibility that these residents might not portray their true feelings in comments about their local cow shelter. However, the face to face technique has the ability to rapidly collect data from a large number of people with less false reporting than other methods. It is also possible that the respondents were not representative of the Indian public. The sample size was large enough, but the study surveyed only a small sector of the population within six states of India.

However, while this research constituted the first attempt at eliciting attitudes towards cows and gaushalas in these areas of India, it was a brief survey and had implicit bias and limitations. More in-depth ethnographic research will be required to fully examine people’s relationship with these ancient institutions and with cows before drawing conclusions as to their motivations, influences, and beliefs.

## 7. Conclusions

The public attitude towards cows and cow welfare in cow shelters was guided by the overriding concept of the cow as sacred, literally having the status of ‘mother goddess’ in Indian society. Visiting the cow shelters frequently for religious reasons further strengthens this status of the cow. The majority of the respondents in this study believed in the welfare of all cows irrespective of their breeds. Welfare and religious reasons were ranked higher as reasons for the establishment and running of the local cow shelters by the respondents, which symbolises the ‘protectionist conservationism’ approach of the Indian society in the context of this study. The older respondents had a focus on the utilitarian and religious values of the cow shelters, whereas the younger people viewed them as institutions for cow welfare and protection. Reverence for cows and concerns about their welfare in the cow shelters increased with increasing education levels. The patriarchal structure of the Indian society was reflected in the neutral views about cow welfare in shelters shown by females. Higher incomes leading to more frequent visits to cow shelters for religious reasons indicates the status of the cow as an incarnation of the ‘goddess of wealth’ in Hindu mythology [11]. Increased religiosity levels and the Hindu religion were the main reasons for establishing and visiting cow shelters, and there was some evidence of community responsibility towards local Indian cow breeds. Place of residence revealed attitudinal differences towards cows and cow shelters. Rural populations held a utilitarian as well as religious view of cow shelters and reported fewer welfare issues. Increased education levels did not reduce reverence for the cow, but it enabled them to report welfare and cow comfort issues in the shelters.

Key differences in the attitudes of the public towards cows and cow shelters across the demographic profiles delineated in this study need to be understood and incorporated into initiatives to improve the welfare of cows in shelters. This will maximise public engagement to successfully manage the cow shelters with modern scientific concepts of animal welfare-based management in order to perpetuate these unique institutions in a sustainable way. Further studies are needed to assess the knowledge levels of the public about cow welfare. This will reveal more about the dichotomy of thoughts of the Indian public towards cows in the context of religion and animal welfare. Future research should identify and address key welfare issues with a broader range of stakeholders and examine the potential impacts of improvements in cow welfare in the cow shelters.

## Figures and Tables

**Table 1:** Descriptive statistics of public survey for the assessment of attitudes towards cow shelters and cow welfare.

Demographic	Descriptor	No. of Respondents	% of Respondents	Indian National Statistics [44]
Gender	Males	415	50.3	51.47%
	Females	410	49.7	48.53%
Age (years)	18–25	108	13.09	Mean: 27.6
	26–35	195	23.64	
	36–45	195	23.64	
	46–55	170	20.61	
	56–65	98	11.88	
	66 and above	59	7.15	
Religion	Hinduism	788	95.52	
	Islam	14	1.70	80%
	Sikhism	13	1.58	13%
	Judaism	5	0.61	1.9%
	Zoroastrianism	4	0.48	0.4% (others)
	Jainism	1	0.12	
Religiosity	Not religious at all	17	2.06	
	Not very religious	9	1.09	
	Moderately religious	411	49.82	
	Very religious	388	47.03	
Ethnicity	Indo-Aryan	808	97.94	72%
	Dravidian	2	0.24	25%
	Others	15	1.82	3%
Education level	No formal education	108	13.09	
	Under grade 10	225	27.27	41.3%
	Grade 10	161	19.52	8.74%
	Grade 12	128	15.52	6.43%
	Diploma	19	2.30	0.59%
	Graduand	118	14.30	Graduand and above—3.47%
	Post-graduand	66	8.00	
Marital status	Single	85	10.30	
	Married	705	85.45	
	Widowed	35	4.24	
No. of children	No children	111	13.45	
	One	107	12.97	
	Two	312	37.82	
	Three	171	20.73	
	Four	82	9.94	
	Five or more	42	5.09	
Annual Income (INR)	<10,000	38	4.61	
	10,000–25,000	112	13.58	
	25,001–50,000	105	12.73	
	50,001–75,000	116	14.06	
	75,001–100,000	135	16.36	
	100,001–500,000	218	26.42	
	500,001–1000,000	52	6.30	
	1,000,001–5,000,000	31	3.76	
	5,000,001–10,000,000	10	1.21	
	>10,000,000	8	0.97	
Place of residence	Village	580	70.30	(Rural) 68.85% 31.15%
	Urban	177	21.45	
	Suburban	46	5.58	
	Country town	22	2.67	

**Table 2:** Respondents’ awareness of, and relationship with gaushalas, and their attitudes to the welfare of cows in gaushalas.

Question	Descriptor	Number of Responses	% Responses
Contact with Cows at Home or Nearby as a Child?	Yes	767	92.97
	No	58	7.03
Are you aware of the gaushala existing nearby?	Yes	821	99.5
	No	4	0.48
How often you visit your local gaushala?	Daily	203	24.61
	Weekly	193	23.39
	Fortnightly	43	5.21
	Monthly	151	18.30
	Every 6 months	105	12.73
	Yearly	44	5.33
	<once a year	14	2.91
	Never visited	62	7.52
Why do you visit gaushalas?	Religious reasons	534	64.73
	Examine cow welfare	100	12.12
	Feed the cows	97	11.76
	Leisure/enjoy seeing cows	81	9.82
	Educational reasons	9	1.09
	Buy cow products	4	0.48
What is best for unwanted cows?	Sheltered in gaushalas	818	99.15
	Export to neighbouring countries	4	0.48
	Slaughter	2	0.24
	Left roaming on the streets	1	0.12
On your gaushala visit, which is your favourite type of cow?	All are favourites	541	65.57
	Local Indian breeds	273	33.09
	Jersey	5	0.60
	Holstein	4	0.48
	Cross breeds	2	0.24
Community responsibility to cow breed types?	Equal to all cows	631	76.48
	More for local breeds	193	23.39
	More for exotic breeds	1	0.12
How important is it for cows to be sheltered in gaushalas? On a scale of 1 to 5 (1, strongly unimportant—5, strongly important)	Strongly unimportant	7	0.85
	Unimportant	6	0.73
	Neither unimportant nor important	20	2.42
	Important	33	4.00
	Strongly important	759	92.0
To what extent do you agree that cows should be kept in gaushalas? (1, strongly agree to 5, strongly disagree)			
Tradition/culture	Strongly disagree	175	21.45
	Disagree	70	8.48
	Neither agree nor disagree	19	2.30
	Agree	384	46.55
	Strongly agree	177	21.45
Animal welfare	Strongly disagree	195	23.64
	Disagree	21	2.55
	Neither agree nor disagree	10	1.21
	Agree	359	47.15
	Strongly agree	210	25.45
Breeding cows	Strongly disagree	99	12.00
	Disagree	393	47.64
	Neither agree nor disagree	107	12.97
	Agree	214	25.94
	Strongly agree	12	1.45
Milk production	Strongly disagree	92	11.15
	Disagree	416	50.42
	Neither agree nor disagree	98	11.88
	Agree	202	24.48
	Strongly agree	17	2.06
How many cows should be housed in your local gaushalas for acceptable animal welfare?	<50	10	1.21
	51–100	47	5.70
	101–150	70	8.50
	151–200	41	4.98
	201–500	56	6.80
	501–1000	31	3.76
	>1000	62	7.52
	According to space available	502	60.92
On a scale of 1–5 (1, strongly unimportant to 5, strongly important), do you feel the gaushala near you provides adequate			
Shelter for the cows	Strongly disagree	5	0.61
	Disagree	32	3.88
	Neither agree nor disagree	82	9.94
	Agree	169	20.48
	Strongly agree	537	65.09
Food and water	Strongly disagree	4	0.48
	Disagree	17	2.06
	Neither agree nor disagree	91	11.03
	Agree	159	19.27
	Strongly agree	554	67.15
Freedom to move about and socialise with other cows	Strongly disagree	5	0.61
	Disagree	34	4.12
	Neither agree nor disagree	67	8.12
	Agree	174	21.09
	Strongly agree	545	66.06
Bedding, flooring and facility for cows to lie down	Strongly disagree	6	0.73
	Disagree	37	4.48
	Neither agree nor disagree	85	10.30
	Agree	187	22.67
	Strongly agree	510	61.82
Humane treatment of the cows	Strongly disagree	6	0.73
	Disagree	13	1.58
	Neither agree nor disagree	109	13.21
	Agree	172	20.85
	Strongly agree	525	63.64
Veterinary care	Strongly disagree	3	0.36
	Disagree	19	2.30
	Neither agree nor disagree	116	14.06
	Agree	189	22.91
	Strongly agree	498	60.36
Do you support your local gaushala?	Yes	822	99.63
	No	3	0.37
Do you have any issues with your local gaushala?	Yes	104	12.61
	No	721	87.39

**Table 3:** Significant effects (*p* < 0.05) of age on public perception about cow welfare and gaushalas in India.

Criterion	Coefficient	SE Coefficient	*p*-Value	OR	95% CI
Rank of importance of the different purposes of establishing gaushalas					
Animal welfare	0.17	0.059	0.003	1.19	1.06–1.34
Milk sales	0.30	0.063	<0.001	1.35	1.20–1.53
Breeding cows	−0.12	0.062	0.04	0.88	0.78–1.00
Attracting funding	−0.13	0.065	0.03	0.87	0.77–0.99
Earning a profit	−0.28	0.069	<0.001	0.75	0.66–0.87
Reasons for keeping cows in gaushalas					
Animal welfare	0.18	0.058	0.002	1.20	1.07–1.35
Breeding cows	0.12	0.058	0.03	1.13	1.01–1.27
Culture/tradition	0.16	0.058	0.005	1.18	1.05–1.32
Provision of shelter by gaushalas is adequate	−0.16	0.068	0.01	0.85	0.74–0.97

**Table 4:** Education level effects on public perception about cow welfare and gaushalas in India (*p* < 0.05).

Criterion	Coefficient	SE Coefficient	*p*-Value	OR	95% CI
Frequency of visiting the local gaushala	−0.13	0.042	0.001	0.87	0.80–0.95
Rank of importance of the purpose of establishing gaushalas					
Animal welfare	0.13	0.045	0.003 *	1.14	1.05–1.25
Milk sales	0.09	0.047	0.03	1.10	1.00–1.21
Breeding cows	−0.14	0.048	0.002	0.86	0.78–0.95
Religious purposes	−0.09	0.044	0.02	0.91	0.83–0.99
Reasons for keeping cows in gaushalas (1 strongly agree to 5 strongly disagree)					
Milk sales	0.09	0.044	0.03	1.10	1.01–1.20
Provision of resources by gaushalas is adequate (1 strongly disagree to 5 strongly agree)					
Bedding, flooring and lying down	0.12	0.047	0.006	1.14	1.04–1.25
Humane treatment	0.10	0.048	0.03	1.11	1.01–1.22
Veterinary care	−0.37	0.113	0.001	0.69	0.55–0.86
Reason of visit to gaushalas					
Religious reasons	0.43	0.100	<0.001	1.54	1.27–1.88

**Table 5:** Gender effects on public perception about cow welfare and gaushalas in India (*p* < 0.05).

Criterion	Parameter	Mean	Coefficient	SE Coefficient	*p*-Value	OR	95% CI
Frequency of visiting the local gaushala (1 daily, 2 weekly, 3 fortnightly, 4 monthly, 5 biennially, 6 annually, 7< annually, 8 never)	Referent: Female	3.71					
	Male	3.01	0.63	0.132	≤0.0001	1.89	1.46–2.45
Importance of gaushalas for cows (1 strongly unimportant—5 strongly important)	Referent: Female	4.90					
	Male	4.80	0.86	0.303	0.004	2.38	1.32–4.32
Reasons for keeping cows in gaushalas (1 strongly agree to 5 strongly disagree)							
Milk sales	Referent: Female	3.58					
	Male	3.30	0.39	0.139	0.004	1.49	1.13–1.95
Breeding cows	Referent: Female	3.54					
	Male	3.30	0.24	0.137	0.07	1.28	0.98–1.68
Provision of resources by gaushalas is adequate (1 strongly disagree to 5 strongly agree)							
Freedom to move about and socialise with other cows	Referent: Female	4.42					
	Male	4.53	-0.34	0.156	0.02	0.71	0.52–0.96
Humane treatment	Referent: Female	4.39					
	Male	4.51	-0.33	0.152	0.02	0.71	0.53–0.96
Reason for visiting gaushala (select most important—Religious, Feed the cows, Educational, Examine welfare, Leisure, Buy products or other)	Referent: Female	1.92					
	Male	2.05	0.99	0.342	0.004	2.70	1.38–5.29

**Table 6:** Religion effects on public perception about cow welfare and gaushalas in India (*p* < 0.05).

Criterion	Parameter	Mean	Coefficient	SE Coefficient	*p*-Value	OR	95% CI
Rank of importance of the purpose of establishing gaushalas (1 most important to 6 least important)							
Earning a profit	Referent: Hinduism	4.87					
	Others	3.94	1.29	0.336	<0.001	3.64	1.88–7.05
Religious purposes	Referent: Hinduism	2.33					
	Others	2.97	−0.86	0.318	0.006	0.42	0.22–0.78
Reasons for keeping cows in gaushalas (1 strongly agree—5 strongly disagree)							
Milk sales	Referent: Hinduism	3.46					
	Others	3.00	0.68	0.322	0.03	1.97	1.05–3.71
Provision of resources by gaushalas is adequate (1 strongly disagree to 5 strongly agree)							
Shelter	Referent: Hinduism	4.44					
	Others	4.70	−0.96	0.440	0.02	0.38	0.16–0.91
Bedding, flooring, and lying down	Referent: Hinduism	4.39					
	Others	4.62	−0.91	0.416	0.02	0.40	0.18–0.91

**Table 7:** Religiosity effects on public perception about cow welfare and gaushalas in India (*p* < 0.05).

Criterion	Coefficient	SE Coefficient	*p*-Value	OR	95% CI
Frequency of visiting the local gaushala (1 daily, 2 weekly, 3 fortnightly, 4 monthly, 5 biannually, annually, 6 <annually, 7 never)	0.27	0.102	0.008	1.31	1.07–1.60
Rank of importance of the purposes of establishing gaushalas (1 most important to 6 least important)					
Earning a profit	−0.28	0.122	0.01	0.75	0.59–0.95
Religious purposes	0.41	0.107	<0.001	1.51	1.22–1.86
Provision of resources by gaushalas is adequate (1 strongly disagree to 5 strongly agree)					
Shelter	−0.27	0.117	0.02	0.76	0.61–0.96
Freedom to move about and socialise with other cows	−0.28	0.117	0.01	0.76	0.60–0.95
Bedding, flooring and lying down	−0.27	0.114	0.01	0.76	0.61–0.95
Humane treatment	−0.34	0.114	0.002	0.71	0.56–0.88
Veterinary care	−0.37	0.113	0.001	0.69	0.55–0.86

**Table 8:** Place of residence effects on public perception about cow welfare and gaushalas in India (*p* < 0.05).

Criterion	Parameter	Mean	Coefficient	SE Coefficient	*p*-Value	OR	95% CI
Frequency of visiting the local gaushala (1 daily, 2 weekly, 3 fortnightly, 4 monthly, 5 biannually, annually, 6 <annually, 7 never)	Referent: Village	3.55					
	Urban	2.63	1.09	0.177	<0.001	3.00	2.12–4.25
Importance of gaushalas for cows (1 strongly unimportant to 5 strongly important)	Referent: Village	4.84					
	Country town	4.45	1.4	0.516	0.004	4.48	1.63–12.33
Rank of importance of the purposes of establishing gaushalas (1 most important to 6 least important)							
Animal welfare	Referent: Village	2.49					
	Urban	1.94	0.57	0.183	0.002	1.78	1.25–2.56
	Suburban	1.69	0.75	0.310	0.015	2.12	1.15–3.90
	Country town	5.05	−3.01	0.476	<0.001	0.05	0.02–0.12
Milk sales	Referent: Village	3.35					
	Suburban	3.10	0.67	0.319	0.03	1.96	1.05–3.66
	Country town	2.25	2.49	0.495	<0.001	12.09	4.58–31.90
Breeding cows	Referent: Village	3.62					
	Suburban	3.93	−0.73	0.325	0.02	0.48	0.25–0.91
	Country town	2.06	3.64	0.514	<0.001	38.37	13.99–105.24
Attracting funding	Referent: Village	4.30					
	Urban	4.63	−0.44	0.200	0.02	0.64	0.43–0.95
	Suburban	4.97	−1.14	0.333	0.001	0.32	0.17–0.61
	Country town	2.43	2.47	0.486	<0.001	11.85	4.56–30.78

**Table 9:** Word frequency count of the question ‘What do you mean by the term welfare of cows?’.

Column Header	Word	Count	Weighted Percentage (%)	Similar Words
1	care	369	17.35	care, cared, caring
2	goddess	316	14.86	goddess, goddesses
3	mother	314	14.76	mother, mothers
4	proper	313	14.72	proper, properly
5	feeding	176	8.27	feeding
6	rescue	71	3.34	rescue, rescued
7	abandoned	49	2.30	abandoned, abandoning, abandonment
8	slaughter	34	1.60	slaughter
9	welfare	29	1.36	welfare
10	duty	27	1.27	duty
11	religion	26	1.22	religion
12	sheltering	26	1.22	shelter, sheltered, sheltering, shelters
13	human	23	1.08	human, humane, humanity, humans
14	religious	22	1.03	religious
15	watering	20	0.94	watering
16	creatures	15	0.71	creature, creatures
17	dumb	13	0.61	dumb
18	Hindu	10	0.47	Hindu
19	worship	10	0.47	worship
